# Combination of FDG-PET and FMISO-PET as a treatment strategy for patients undergoing early-stage NSCLC stereotactic radiotherapy

**DOI:** 10.1186/s13550-019-0578-6

**Published:** 2019-12-04

**Authors:** Shiro Watanabe, Tetsuya Inoue, Shozo Okamoto, Keiichi Magota, Ayumi Takayanagi, Jun Sakakibara-Konishi, Norio Katoh, Kenji Hirata, Osamu Manabe, Takuya Toyonaga, Yuji Kuge, Hiroki Shirato, Nagara Tamaki, Tohru Shiga

**Affiliations:** 1 Department of Nuclear Medicine, Hokkaido University Graduate School of Medicine, Kita-15, Nishi-7, Kita-ku, Sapporo, 060-8638 Japan; 2 Department of Radiation Medicine, Hokkaido University Graduate School of Medicine, Kita-15, Nishi-7, Kita-ku, Sapporo, 060-8638 Japan; 3 Department of Radiology, Obihiro Kosei Hospital, West 14 South 10-1, Obihiro, 080-0024 Japan; 4 Division of Medical Imaging and Technology, Hokkaido University Hospital, Kita-14, Nishi-5, Kita-ku, Sapporo, 060-8648 Japan; 5 Department of Diagnostic and Interventional Radiology, Hokkaido University Graduate School of Medicine, Kita-15, Nishi-7, Kita-ku, Sapporo, 060-8638 Japan; 6 First Department of Medicine, Hokkaido University Hospital, Kita-14, Nishi-5, Sapporo, 060-8648 Japan; 7 PET Center, Department of Radiology and Biomedical Imaging, Yale University School of Medicine, New Haven, CT 06520 USA; 8 Central Institute of Isotope Science, Hokkaido University, Kita-15, Nishi-7, Kita-ku, Sapporo, 060-8638 Japan; 9 Department of Radiology, Kyoto Prefectural University of Medicine, Kajii-cho, Kawaramachi-Hirokoji, Kamigyo-ku, Kyoto, 602-8566 Japan

**Keywords:** Non-small cell lung cancer, Hypoxia, Fluoromisonidazole, Fluorodeoxyglucose, Stereotactic body radiation therapy

## Abstract

**Background:**

We investigated the prognostic predictive value of the combination of fluorodeoxyglucose (FDG)- and fluoromisonidazole (FMISO)-PET in patients with non-small cell lung carcinoma (NSCLC) treated with stereotactic body radiation therapy (SBRT).

**Patients and methods:**

We prospectively examined patients with pathologically proven NSCLC; all underwent FDG and FMISO PET/CT scans before SBRT. PET images were acquired using a whole-body time-of-flight PET-CT scanner with respiratory gating. We classified them into recurrent and non-recurrent groups based on their clinical follow-ups and compared the groups' tumor diameters and PET parameters (i.e., maximum of the standardized uptake value (SUVmax), metabolic tumor volume, tumor-to-muscle ratio, and tumor-to-blood ratio). We performed univariate analysis to evaluate the impact of the PET variables on the patients' progression-free survival (PFS). We divided the patients by thresholds of FDG SUVmax and FMISO SUVmax obtained from receiver operating characteristic analysis for assessment of recurrence rate and PFS.

**Results:**

Thirty-two NSCLC patients (19 male and 13 females; median age, 83 years) were enrolled. All received SBRT. At the study endpoint, 23 patients (71.9%) were non-recurrent and nine patients (28.1%) had recurrent disease. Significant between-group differences were observed in tumor diameter and all the PET parameters, demonstrating that those were significant predictors of the recurrence in all patients. In the 22 patients with tumors > 2 cm, tumor diameter and FDG SUVmax were not significant predictors. Thirty-two patients were divided into three patterns from the thresholds of FDG SUVmax (6.81) and FMISO SUVmax (1.89); A, low FDG and low FMISO (*n* = 14); B, high FDG and low FMISO (*n* = 8); C, high FDG and high FMISO (*n* = 10). No pattern A patient experienced tumor recurrence, whereas two pattern B patients (25%) and seven pattern C patients (70%) exhibited recurrence. A Kaplan-Meier analysis of all patients revealed a significant difference in PFS between patterns A and B (*p* = 0.013) and between patterns A and C (*p* < 0.001). In the tumors > 2 cm patients, significant differences in PFS were demonstrated between pattern A and C patients (*p* = 0.002).

**Conclusion:**

The combination of FDG- and FMISO-PET can identify patients with a baseline risk of recurrence and indicate whether additional therapy might be performed to improve survival.

## Background

Stereotactic body radiation therapy (SBRT) has been applied to early-stage non-small cell lung cancer (NSCLC) and was shown to provide a good survival benefit for both operable and inoperable NSCLC cases [1, 2]. However, the 3-year overall survival (OS) rates of patients with stage I NSCLC was higher in patients who were treated with a lobectomy (73%) and only 65% in the patients who were treated with SBRT [3]. The optimal treatment for the local recurrence and metastasis of NSCLC is thus a matter of controversy, and it is important to identify the NSCLC patients who are likely to benefit from SBRT when selecting SBRT candidates [4].

^18^F-fluorodeoxyglucose (FDG) positron emission tomography (PET) has been widely used in malignant tumor preoperative staging work-ups, especially for lung cancer [5]. The uptake of FDG is a potential biomarker for identifying NSCLC patients who are at high risk of recurrence or death [6–9]. The maximum of the standardized uptake value (SUVmax) is an easily obtained and robust parameter, but the SUVmax does not fully reflect the tumor size or tumor heterogeneity. In addition, inflammatory changes and necrosis each have an impact on the FDG uptake.

Intratumoral hypoxia generally accelerates radioresistance and chemoresistance, and thus for hypoxic tumors a 2.5- to 3-fold higher radiotherapy dose is necessary to achieve the same cytotoxic effect [10]. ^18^F-fluoromisonidazole (FMISO) is a major PET tracer for hypoxia imaging, and several research groups have evaluated the potential role of FMISO as a prognostic tool and for the assessment of the presence of tumor reoxygenation after nonsurgical treatment of NSCLC [11].

Though hypoxia may have a great impact on SBRT outcomes because of the lack of reoxygenation that would occur during conventional radiation therapy, few studies have been conducted to clarify the relationship between the existence of hypoxia and the prognosis of NSCLC patients treated with SBRT. We hypothesized that a combination of the measurement of metabolic activity and the measurement of tumor hypoxia status would be useful to stratify the prognoses of NSCLC patients treated with SBRT. We conducted the present study to determine the prognostic predictive value of combined FDG- and FMISO-PET for NSCLC patients treated with SBRT.

## Methods

### Patients

The study was approved by the Institutional Review Board of Hokkaido University (#012-0406). Patients with pathologically confirmed early-stage NSCLC at Hokkaido University Hospital who were under consideration for SBRT and who signed the consent form to undergo FMISO PET/CT were prospectively enrolled from August 2013 to August 2017. None of the patients had ever received radiotherapy. The respiratory status of the patients was not considered as an exclusion criterion. All of the patients were followed-up with basic CT examinations every 3 months for the first 2 years after their treatment and every 6 months thereafter.

Progression-free survival (PFS) was defined as the number of days from the start of treatment until relapse or death due to any cause or the last follow-up date. We used the Response Evaluation Criteria in Solid Tumors 1.1 (RECIST) criteria to define the progressive disease as “relapse”. We classified the patients who did not exhibit relapse based on these criteria as the non-recurrent group, and the patients who relapsed during the follow-up as the recurrent group.

### SBRT procedure

All of the patients received SBRT to lung tumors as the definitive radiotherapy the day after the FMISO-PET was performed. The SBRT for all of the patients was performed with a real-time tumor-tracking radiotherapy (RTRT) system. The RTRT system has been described in detail [12–14]. In brief, 1.5-mm gold markers were implanted near the tumor with bronchoscopy guidance. CT scans were taken with the patient holding his/her breath at the end of normal expiration. RTRT is gated to irradiate the tumor only when the implanted fiducial marker is within 2 mm from its planned position. The gross tumor volume (GTV) was contoured in axial CT images. The clinical target volume (CTV) was considered to be equal to the GTV. The internal target volume (ITV) was three-dimensionally defined as the CTV plus a 3-mm margin based on the gating window. The planning target volume (PTV) was three-dimensionally defined as the ITV plus a 5-mm margin with optimal reduction near the organ at risk (OAR).

The PTV was three-dimensionally defined as the CTV plus a 5-mm margin with optimal reduction near the OAR. Using a superposition algorithm, we administered 48 Gy in four fractions at the isocenter or 40 Gy in four fractions to the 95% volume of the PTV (PTV D95) with a treatment period of 4–7 days. All patients were treated with 6-MV photons. The SBRT was delivered using multiple noncoplanar static ports.

### PET/CT studies

**Fig. 1:**
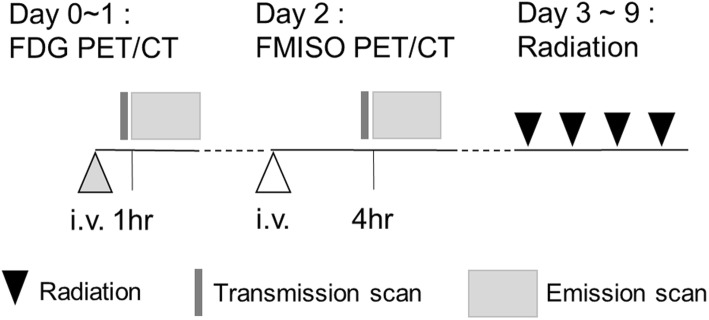
The FDG and FMISO scan and SBRT procedure

PET images were acquired using a whole-body time-of-flight PET-CT scanner (GEMINI-TF; Philips Japan, Tokyo). The PET scanning protocol is illustrated in Fig. 1. Each patient first underwent an FDG PET/CT scan, and then the FMISO PET/CT scan 1 or 2 days later. Before the FDG PET/CT, all patients fasted for ≥ 6 hr (oral hydration with glucose-free water was allowed). For the FDG PET/CT examination, 4.5 MBq/kg of FDG was administered intravenously. At 1 hr after the injection, a respiratory-gated four-dimensional (4D) CT scan was obtained and three single-bed static emission scans with the field of view (FOV) covering the entire thorax were obtained in the 3-D mode, followed by a 10-min list-mode PET acquisition with respiratory gating in one-bed position centered on the primary tumor. A chest CT examination was then performed as the last step in the clinical protocol.

For the patients' FMISO PET/CT examinations, we intravenously administered 400 MBq of FMISO per patient (median 7.2 MBq/kg; interquartile range (IQR), 6.5–8.3 MBq/kg). At 4 hr after the injection, a respiratory-gated 4D CT scan and a 30-min list-mode PET acquisition in one-bed position centered on the primary tumor were obtained. A respiratory gating system (Anzai Medical, Tokyo) was attached to the patient’s upper abdomen to measure the respiratory signal.

For the reconstruction of the respiratory-gated images, the PET list-mode data were retrospectively binned into 5-phase frames between inspirations based on the respiratory signals shown by the Anzai gating system. The third phase, which corresponds to expiration, was used for the reconstruction. For all of the PET image reconstructions, photon attenuation was corrected using 4D CT images. The CT images were reconstructed for attenuation and scatter corrections (3-mm contiguous slices) on a 512 × 512 matrix. PET images were iteratively reconstructed using a 3D blob-based iterative list-mode ordered-subsets expectation maximization (OSEM) algorithm with time-of-flight information and the following default settings: iterations, 3; subsets, 33; blob increment, 2.0375 voxels; blob radius, 2.5 voxels; blob shape parameter alpha, 8.3689; and relaxation parameter, 0.6. The image matrix size was 144 × 144 pixels for the 576-mm FOV, and the voxel size was 4 × 4 × 4 mm. The reconstruction included corrections for normalization, dead time, attenuation, scatter, random coincidences, sensitivity, and decay. The reconstructed images were not additionally post-filtered.

### Image analysis

The SUVmax of each primary tumor was obtained in the transaxial view of the FDG PET/CT images. We measured the metabolic tumor volume (MTV) of each primary tumor by using the adaptive threshold method [15]. The FMISO uptake was quantified using (1) the SUVmax, (2) the tumor-to-muscle ratio (TMR), and (3) the tumor-to-blood ratio (TBR). For the background of the TMR, a 1-cm spherical volume of interest was placed in paravertebral skeletal muscle in the same axial slice with the primary tumor for the quantification of the muscle SUVmax. The regions of interest (ROIs) were manually placed on the uptake in the aortic lumen by a single observer who was an experienced nuclear physician and unaware of the patient's information, in the same axial slice with primary tumor. If aorta was not present in the same plane, ROIs were placed at the aortic arch. The mean of SUV in the ROIs was used for calculating the TBR.

### Statistical analyses

The statistical analyses were performed with the software program JMP® 14 (SAS, Cary, NC, USA). Descriptive data are expressed as the median and IQR. The Mann-Whitney *U* test or Fisher's exact probability test was used for the evaluation of the significance of differences in the patients’ clinical and PET parameters between the groups of non-recurrent and recurrent patients. The optimal thresholds of continuous variables for predicting the patients' responses were determined based on the Youden index calculated from the area under the curve (AUC) using a receiver operating characteristic (ROC) analysis. We divided the patients based on the thresholds of FDG SUVmax and FMISO SUVmax for assessment of recurrence rate and PFS.

Correlations between pairs of variables were calculated with the Spearman correlation coefficient. The PFS was calculated by the Kaplan-Meier method and analyzed by the log-rank test. We performed univariate analyses to evaluate the impact of the PET and the other clinical variables on the patients’ PFS by using a Cox proportional hazards regression model. We used Holm's method [16] to adjust the *p* values of the three groups in the Kaplan-Meier analysis. Due to the small sample size (*n* = 32), multivariate analyses were not performed.

## Results

### The NSCLC patients’ characteristics

A final total of 32 NSCLC patients (19 males and 13 females; median age, 83 years) were enrolled. All received SBRT (40 Gy/4 fr (PTV D95), *n* = 23; 48 Gy/4 fr (isocenter), *n* = 9). The patients' characteristics are summarized in Table 1. At the end of the study, 23 patients (71.9%) were non-recurrent and the other nine (28.1%) had the recurrent disease: local sites only (*n* = 1), regional recurrence (*n* = 5), and distant recurrence (*n* = 4). The median follow-up period in the non-recurrent patients was 662 days (range 98–1746 days). The recurrence rate was 26.1% (6 out of 23) and 33.3% (3 out of 9) in treated by 40 Gy/4 fr (PTV D95) and 48 Gy/4 fr (isocenter), respectively.

**Table 1:** Patients’ characteristics

Characteristic	No.	
Age		
Median (range)	83 (56–89)	
Gender		
Male	19	59.4%
Female	13	40.6%
Histology		
Adenocarcinoma	24	75.0%
Squamous cell carcinoma	7	21.9%
Non-small cell carcinoma	1	3.1%
Morphology		
Solid/part solid	26	81.3%
GGO	6	18.8%
Stage		
I	23	71.9%
II	9	28.1%
Prescription dose:		
40 Gy/4 fr (PTV D95)	23	71.9%
48 Gy/4 fr (isocenter)	9	28.1%
Prognosis		
Non-recurrent	23	71.9%
Recurrent	9	28.1%

*PTV* planning target volume

### The FDG-PET and FMISO-PET imaging parameters

Our comparisons of the non-recurrent and recurrent patients revealed significant differences in the tumor diameter (21.0, 15.0–27.5 mm vs. 28.0, 26.0–35.0 mm, *p* = 0.013), the FMISO SUVmax (1.15, 0.82–1.59 vs. 2.21, 1.90–2.74, *p* < 0.001), the TMR (0.83, 0.68–1.19 vs. 2.22, 1.79–2.44, *p* < 0.001), the TBR (0.75, 0.60–1.04 vs. 1.70, 1.37–1.80, *p* = 0.001), and the FDG SUVmax (6.02, 1.81–8.94 vs. 9.73, 8.46–13.21, *p* = 0.005) (Table 2). No significant between-group difference was detected in patient age, gender, pathology (adeno vs. non-adeno), morphology (ground-glass opacity vs. part solid/solid), prescription dose, or the MTV. The tumor diameter and the other PET parameters were significantly correlated with each other (Spearman correlation range, 0.358–0.488). Spearman's correlation coefficients are listed in Table 3.

**Table 2:** Non-recurrent vs. recurrent group

	All patients	Non-recurrent	Recurrent	*p* value
Age (*n*)	32	23	9	
Median, IQR	83, 79–85	83, 79–85	82, 81–85	0.916
Gender				
Female	13	9	4	1.000
Male	19	14	5	
Histology				
Ad	24	18	6	0.655
Non-Ad	8	5	3	
Morphology				
Solid/part solid	26	17	9	0.150
GGO	6	6	0	
Dose				
40 Gy (PTV D95)	23	17	6	0.685
48 Gy (isocenter)	9	6	3	
Tumor diameter	23.0, 17.0–32.3	21.0, 15.0–27.5	28.0, 26.0–35.0	0.013
FDG SUVmax	7.89, 2.00–10.1	6.02, 1.81–8.94	9.73, 8.46–13.2	0.005
FDG MTV	6.62, 1.15–10.0	6.21, 3.71–9.50	8.19, 3.52–10.8	0.586
FMISO SUVmax	1.50, 0.88–2.05	1.15, 0.82–1.59	2.21, 1.90–2.74	< 0.001
FMISO TMR	1.11, 0.72–1.80	0.83, 0.68–1.19	2.22, 1.79–2.44	< 0.001
FMISO TBR	0.91, 0.63–1.41	0.75, 0.60–1.04	1.70, 1.37–1.80	< 0.001

*FDG* fluorodeoxyglucose, *FMISO* fluoromisonidazole, *IQR* interquartile range, *MTV* metabolic tumor volume, *PTV* planning target volume, *SUVmax* maximum of standardized uptake value, *TBR* tumor-to-blood ratio, *TMR* tumor-to-muscle ratio

**Table 3:** Spearman’s correlation coefficients

All patients (n = 32)	FDG SUVmax		FMISO SUVmax		FMISO TMR		FMISO TBR	
Tumor diameter	0.363	*p* = 0.041	0.488	*p* = 0.005	0.439	*p* = 0.012	0.358	*p* = 0.044
FDG SUVmax			0.798	*p* < 0.001	0.756	*p* < 0.001	0.729	*p* < 0.001
FMISO SUVmax					0.959	*p* < 0.001	0.940	*p* < 0.001
FMISO TMR							0.961	*p* < 0.001
Larger tumor (n = 22)	FDG SUVmax		FMISO SUVmax		FMISO TMR		FMISO TBR	
Tumor diameter	0.201	*p* = 0.369	0.213	*p* = 0.342	0.162	*p* = 0.472	0.083	*p* = 0.714
FDG SUVmax			0.751	*p* < 0.001	0.700	*p* < 0.001	0.635	*p* = 0.002
FMISO SUVmax					0.893	*p* < 0.001	0.922	*p* < 0.001
FMISO TMR							0.949	*p* < 0.001

Abbreviations are explained in Table 2 footnote

We analyzed the subgroup of patients whose tumor diameter was ≥ 20 mm (*n* = 22) as the larger-tumor group because all of the patients with a tumor < 20 mm were in the non-recurrent group. In the larger-tumor group, the tumor diameter had no correlation with any of the PET parameters, but the PET parameters showed significant correlations with each other (Spearman correlation range, 0.635–0.949, Table 3). As shown in Table 3, in the larger-tumor group, there was no significant difference in tumor diameter between the non-recurrent and recurrent patients (26.0, 22.0–33.0 mm vs. 28.0, 26.0–35.0 mm, *p* = 0.33), but there were significant differences between the non-recurrent and recurrent patients in the FMISO SUVmax (1.42, 0.94–1.67 vs. 2.21, 1.90–2.74, *p* < 0.01), the TMR (1.07, 0.72–1.54 vs. 2.22, 1.79–2.44, *p* < 0.01), the TBR (0.91, 0.63–1.12 vs. 1.70, 1.37–1.80, *p* < 0.01), and the FDG SUVmax (6.98, 5.38–9.79 vs. 9.73, 8.46–13.21, *p* = 0.02) (Table 4).

**Table 4:** Non-recurrent vs. recurrent patients in the larger-tumor group (*n* = 22)

	All 22 patients	Non-recurrent (*n* = 13)	Recurrent (*n* = 9)	*p* value
Tumor diameter	27.0, 23.0–34.0	26.0, 22.0–33.0	28.0, 26.0–35.0	0.331
FDG SUVmax	8.59, 6.17–11.8	6.98, 5.38–9.79	9.73, 8.46–13.21	0.015
FDG MTV	8.06, 5.09–10.42	6.60, 1.89–9.15	8.09, 3.52–10.8	0.973
FMISO SUVmax	1.70, 1.41–2.32	1.42, 0.94–1.67	2.21, 1.90–2.74	0.002
FMISO TMR	1.45, 1.05–1.94	1.07, 0.72–1.54	2.22, 1.79–2.44	0.001
FMISO TBR	1.13, 0.81–1.49	0.91, 0.63–1.12	1.70, 1.37–1.80	0.002

Abbreviations are explained in Table 2 footnote

### The patients' prognoses

We used a logistic regression analysis to examine the tumor diameter and the PET parameters of FDG SUVmax, FMISO SUVmax, TMR, and TBR. The analysis results demonstrated that all of these PET parameters and the tumor diameter were significant predictors of the patients' response to SBRT in all 32 patients (Table 5). In the larger-tumor group, the tumor diameter and the FDG SUVmax were not significant predictors. The FMISO parameters were significant predictors of the SBRT response (Table 5).

**Table 5:** Results of Cox regression analysis/univariate analysis

Variable	HR	95% CI	*p*
All patients			
Tumor diameter	1.107	1.020–1.201	0.014
FDG SUVmax	1.108	1.007–1.220	0.035
FMISO SUVmax	4.374	1.726–11.08	0.002
FMISO TMR	5.670	1.973–16.29	0.001
FMISO TBR	9.309	2.371–36.54	0.001
Larger-tumor patients			
Tumor diameter	1.06	0.960–1.172	0.251
FDG SUVmax	1.08	0.980–1.191	0.121
FMISO SUVmax	3.628	1.367–9.264	0.010
FMISO TMR	4.745	1.552–14.50	0.006
FMISO TBR	12.71	2.218–72.87	0.004

Abbreviations are explained in Table 2 footnote

**Fig. 2:**
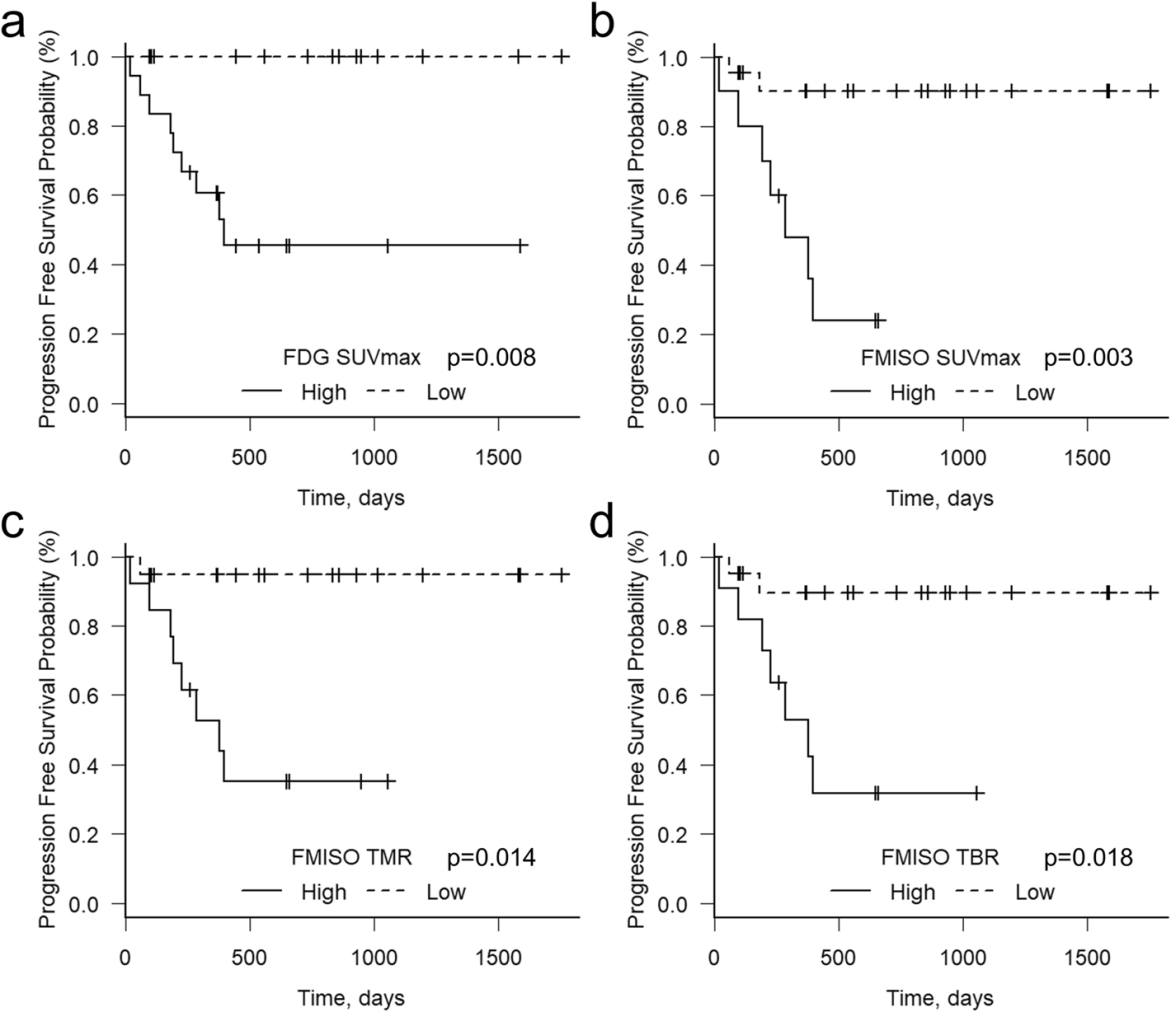
The PFS of the total series of 32 patients stratified by their values of FDG SUVmax (6.91), FMISO SUVmax (1.89), TMR (1.25), and TBR (1.14). The PFS of the different subgroups divided by FDG SUVmax (*p* = 0.008), FMISO SUVmax (*p* = 0.003), TMR (*p* = 0.014), and TBR (*p* = 0.018) differed significantly. FDG fluorodeoxyglucose, FMISO fluoromisonidazole, PFS progression-free survival, SUVmax maximum of standardized uptake value, TBR tumor-to-blood ratio, TMR tumor-to-muscle ratio

The parameter thresholds defined by the ROC curve analysis were as follows: FDG SUVmax, 6.91; FMISO SUVmax, 1.89; TMR, 1.25; and TBR, 1.14. The PFS of the patient subgroups divided by the FDG SUVmax (*p* = 0.008), FMISO SUVmax (*p* = 0.003), TMR (*p* = 0.014), and TBR (*p* = 0.018) were significantly different in the Kaplan-Meier analysis (Fig. 2).

### The patterns of FDG SUVmax and FMISO SUVmax

**Fig. 3:**
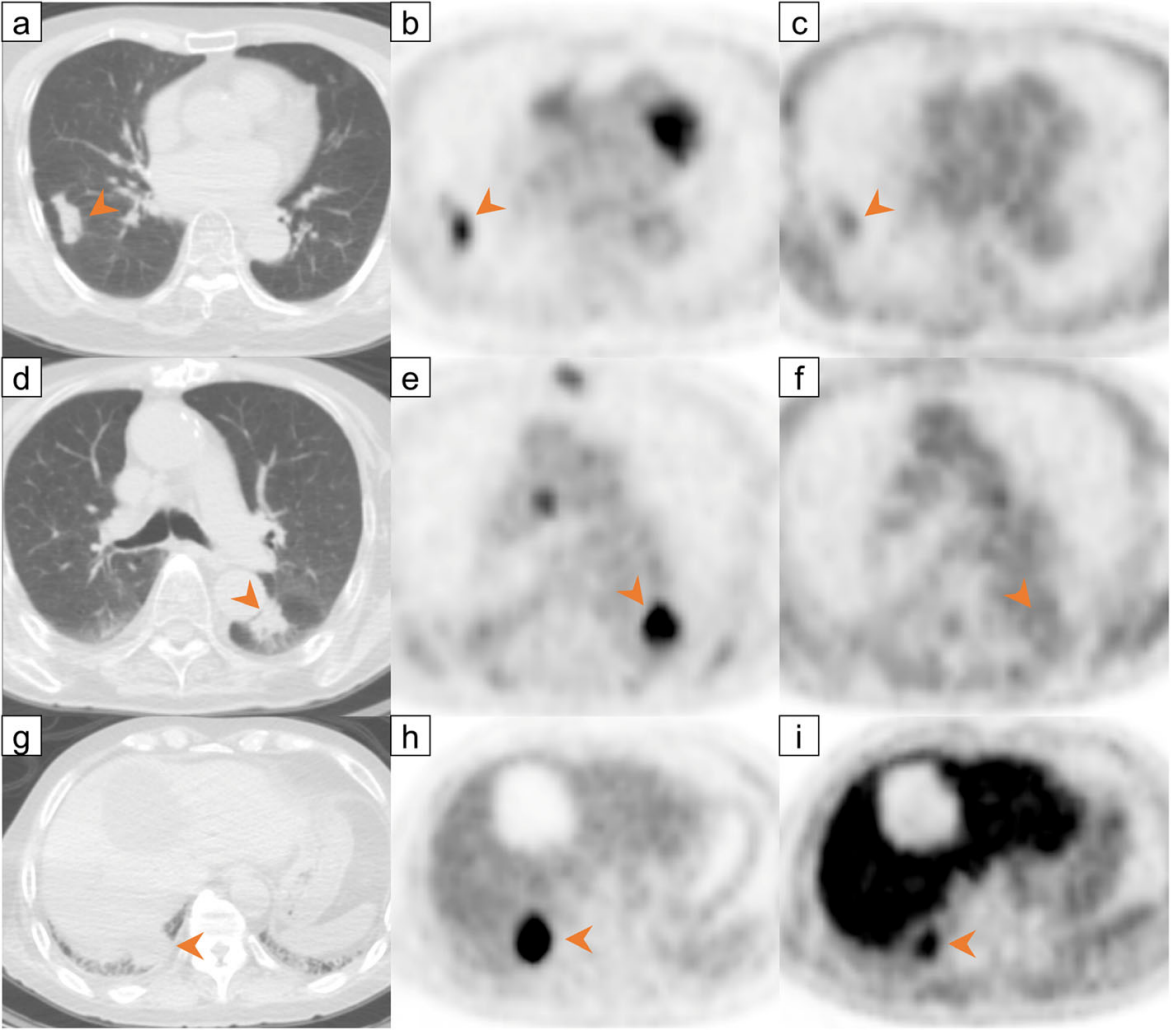
Representative cases of the three patterns of FDG and FMISO SUVmax values. **a** CT, **b** FDG PET (SUVmax = 6.02). **c** FMISO PET (SUVmax = 1.54) image of a patient with pattern A (both low FDG and low FMISO SUVmax) who had no evidence of recurrence on follow-up at 949 days after treatment completion. **d** CT, **e** FDG PET (SUVmax = 8.73). **f** FMISO PET (SUVmax = 1.42) image of a patient with pattern B (high FDG SUVmax and low FMISO SUVmax) who had no evidence of recurrence on follow-up at 540 days after treatment completion. **g** CT, **h** FDG PET (SUVmax = 14.04). **i** FMISO PET (SUVmax = 3.36) image of a patient with pattern C (both high FDG and high FMISO SUVmax) who had recurrence on a mediastinum lymph node on follow-up CT at 195 days after treatment completion

**Fig. 4:**
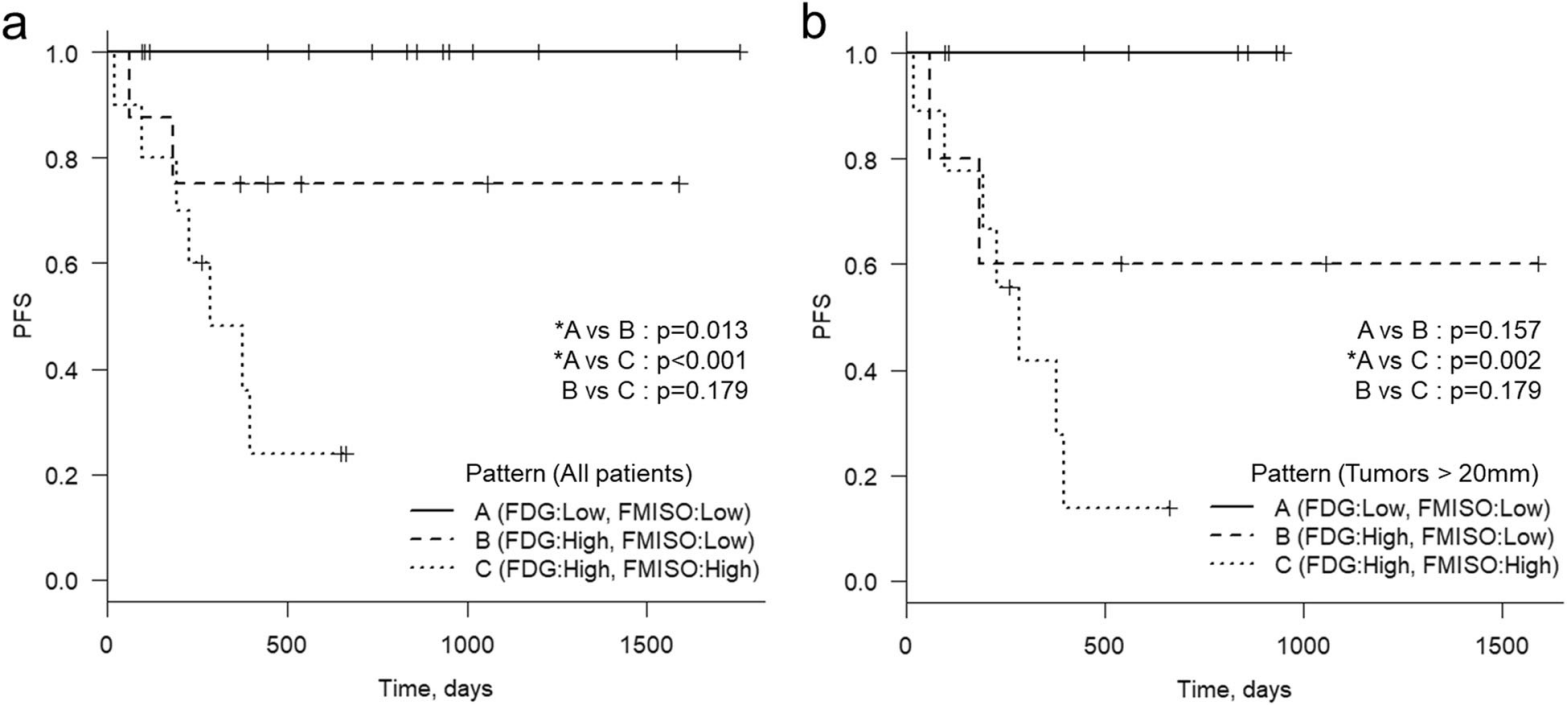
PFS values stratified by the patterns of FDG and FMISO SUVmax. **a** The PFS of all 32 patients according to their FDG and FMISO SUVmax values (6.91 and 1.89, respectively). **b:** The PFS of the 22 patients whose tumor diameter was > 20 mm, based on their FDG and FMISO SUVmax values (6.91 and 1.89, respectively)

Thirty-two patients were divided into three patterns based on the thresholds of FDG SUVmax (6.81) and FMISO SUVmax (1.89), as follows. Pattern A, both low FDG and low FMISO (*n* = 14); pattern B, high FDG and low FMISO (*n* = 8); pattern C, both high FDG and high FMISO (*n* = 10) (Table 6, Fig. 3). No patients were “low FDG and high FMISO.” None of the patients with pattern A developed tumor recurrence, whereas two of the eight (25%) patients with pattern B and seven of the 10 (70%) patients with pattern C developed recurrence. The Kaplan-Meier analysis of the PFS of all 32 patients revealed a significant difference between the pattern A and pattern B patients (*p* = 0.013) and between the pattern A and pattern C patients (*p* < 0.001). In the larger-tumor group, significant differences in PFS were demonstrated between the pattern A and pattern C patients (*p* = 0.002) (Fig. 4).

**Table 6:** Prognosis by FDG and FMISO SUVmax pattern

Pattern	A	B	C
FDG	Low	High	High
FMISO	Low	Low	High
Non-recurrent	14	6	3
Recurrent	0	2	7

Abbreviations are explained in Table 2 footnote

## Discussion

Because a growing number of patients with early-stage NSCLC are being treated with SBRT, it is necessary to evaluate the patients’ treatment response and to predict the outcomes as soon as possible in order to provide the optimal treatment to improve survival. PET is a promising modality for this purpose in NSCLC. In this study, we evaluated the relationships among metabolic activity, the existence of hypoxia, and the prognoses of early-stage NSCLC patients who underwent SBRT. The results of our analyses demonstrated that the activity of glucose metabolism and intracellular hypoxia in the primary tumor of early-stage NSCLC, as measured by FDG and FMISO PET, was associated with a shorter PFS. This finding suggests that since FDG and FMISO uptake are indicators of the poor prognostic potential of NSCLC, patients who are being considered for SBRT should be stratified by their baseline metabolic and hypoxic status.

Our findings also provide clinical evidence of the negative prognostic values of tumor metabolic activity and hypoxia among patients with NSCLC, in agreement with published data [17–20]. FDG PET has been essential for staging and treatment assessments, and it provides additional information concerning the biological characteristics of tumors. In a recent meta-analysis, patients with high FDG SUVmax values in the primary tumor before SBRT showed short overall survival, poor local control, and frequent distant metastasis [21]. However, there is an overlap of FDG SUV values between “good” and “poor” prognosis groups. In addition, the FDG uptake and/or the SUV may fluctuate for multiple reasons; for example, the patient's blood glucose level, fasting quality, FDG excretion quantity, lean/fat mass difference, and residual respiratory motion artifact. It is difficult to select a treatment strategy based on the patient's FDG SUV in daily clinical practice. In our present investigation, the FDG uptake was a significant predictor of short PFS in all patients but was not significant in the patients whose tumor was > 2 cm. This finding indicated that FDG PET may not be able to predict the prognosis well in patients with large tumors.

However, although FMISO has slow pharmacokinetics and must be evaluated 3–4 hr after its administration, FMISO PET requires no preparation and provides a relatively robust evaluation [22]. Cherk et al. prospectively studied 17 NSCLC patients who underwent both FMISO PET and FDG PET, and they reported that the FMISO uptake showed no correlation with the FDG uptake (*r* = 0.26) [23]. Another study of eight NSCLC patients treated with a combination of chemotherapy and/or radiation therapy of 50.0 Gy in 2.0-Gy fractions who underwent FDG PET and FMISO PET scans indicated that a decrease in uptake after treatment was associated with favorable outcome, and a high initial FMISO uptake was a poor prognostic indicator and was not associated with the treatment response [24].

In the largest series of patients with NSCLC in a multicenter and prospective study to date, Vera et al. demonstrated that the probability of disease-free survival was significantly lower in their FMISO-positive patients, regardless of the radiotherapy dose (i.e., whether 66 Gy or more) [25]. We also observed herein that the FMISO uptake was a significant predictor of short PFS, (in patients with tumors > 2 cm). As patients with more hypoxic tumors may achieve only a treatment failure, the hypoxic status of individual tumors should be considered when designing radiation therapy, especially in tumors > 2 cm and those with a high FDG uptake.

Among our present NSCLC patients, those with pattern C (high FDG and high FMISO) had the poorest prognosis. Since FDG uptake has been correlated with the tumor growth rate, the proliferation capacity, and aggressiveness [26], it is not surprising that a higher FDG uptake reflected biologic aggressiveness and poor prognosis in those patients [26]. Similarly, in our present study, higher FDG SUVmax values (18 of the 32 patients, 56.3%) were significantly associated with poor PFS. In addition, a higher FMISO SUVmax value (shown by 10 of the 32 patients, 31.3%) was also an important predictor of the response to SBRT. Fifty percent (9 of 18) of our patients who showed high FDG were recurrent, and 70% (7/10) of the patients who showed a high FMISO SUVmax were recurrent.

In light of the increasing use of SBRT for early-stage NSCLC, a landmark for adjuvant decision-making is needed to identify alternative predictive biomarkers that can be used to stratify patients by their risk of recurrence at a distant site. Hypoxic cancer cells are radioresistant, and it was reported that in nasopharyngeal carcinoma the FMISO uptake in recurrent regions was significantly higher than that in non-recurrent regions [27]. Among our patients, local failure and pleural dissemination were seen in only one patient (3.1%) respectively, whereas lymph node metastasis and distant metastases were seen in five and four patients (15.6% and 12.5%), respectively. Lymph node and distant metastases were not detected in the pretreatment diagnostic imaging. Adjuvant chemotherapy was demonstrated to reduce the distant relapse in surgical resection [28] and SBRT [29, 30]. We speculate that micrometastasis may have already occurred before those patients’ SBRT, even though imaging modalities cannot detect it. Since hypoxia is a potent microenvironmental factor that promotes metastatic progression [31], patients with hypoxic NSCLC might more frequently have micrometastasis.

Based on our findings, FMISO PET may have a potential role as a biomarker for identifying patients who are at a higher risk of recurrence or death. It may also be able to use FMISO PET to differentiate candidates for future trials of additional systemic therapy such as chemotherapy or an immune-checkpoint inhibitor.

Our study’s prospective nature, investigation of a standard hypoxia radiotracer, and homogenous cohort are its major strengths. The data we obtained could be the basis of the relationship between hypoxia and SBRT in NSCLC. The major limitations of our study were the limited sample size (*n* = 32), the single-center design, and the lack of long follow-up and overall survival data. We did not evaluate the optimal thresholds of imaging hypoxia or the patients' outcomes. The number of clinical studies related to the treatment effects of chemoradiotherapy and hypoxia is still very limited. Although Li et al. explored representative hypoxia parameters shown by FMISO PET that could be used to predict the treatment response and prognosis of patients treated with chemoradiotherapy [32], larger and multicenter studies should be designed to test our present findings and to optimize the hypoxia thresholds in NSCLC patients.

## Conclusion

Pretherapy metabolic activity and the hypoxic state in patients with early-stage NSCLC are a strong predictor for recurrence after SBRT. In particular, for large-size tumors, FDG did not show good prognostic power, but FMISO did. The combination of FDG PET and FMISO PET can identify patients with a baseline risk of early recurrence and whether additional therapy should be performed to improve survival. Further prospective studies are warranted to optimize the PET protocol and the thresholds of FDG and FMISO uptake in multicenter studies.

## Data Availability

The datasets used and/or analyzed during the current study are available from the corresponding author on reasonable request.

## Abbreviations

CTV: Clinical target volume
FDG: Fluorodeoxyglucose
FMISO: Fluoromisonidazole
GTV: Gross tumor volume
IQR: Interquartile range
ITV: Internal target volume
MTV: Metabolic tumor volume
NSCLC: Non-small cell lung carcinoma
OAR: Organ at risk
PET: Positron emission tomography
PFS: Progression-free survival
PTV: Planning target volume
RTRT: Real-time tumor-tracking radiotherapy
SBRT: Stereotactic body radiation therapy
SUVmax: Maximum of standardized uptake value
TBR: Tumor-to-blood ratio
TMR: Tumor-to-muscle ratio
